# Transcriptomic Analysis Provides New Insights into Oocyte Growth and Maturation in Greater Amberjack (*Seriola dumerili*)

**DOI:** 10.3390/ani15030333

**Published:** 2025-01-24

**Authors:** Jiahui Yang, Xiaoying Ru, Yang Huang, Jinhui Wu, Tonglin Yang, Peipei Chen, Jin Li, Kunfeng Zhu, Chunhua Zhu

**Affiliations:** 1 Guangdong Research Center on Reproductive Control and Breeding Technology of Indigenous Valuable Fish Species, Guangdong Provincial Key Laboratory of Aquatic Animal Disease Control and Healthy Culture, Fisheries College of Guangdong Ocean University, Zhanjiang 524088, China; 2112201069@stu.gdou.edu.cn (J.Y.); zjouhy@126.com (Y.H.); ytl990617@163.com (T.Y.); 19937071305@163.com (P.C.); 19570035415@163.com (J.L.); 2112101067@stu.gdou.edu.cn (K.Z.); 2 Development and Research Center for Biological Marine Resources, Southern Marine Science and Engineering Guangdong Laboratory (Zhanjiang), Zhanjiang 524025, China; ruxiaoying@zjblab.com; 3 Agro-Tech Extension Center of Guangdong Province, Guangzhou 510500, China; wjhin@sina.com

**Keywords:** *Seriola dumerili*, different developmental stages, ovary, transcriptomic analysis

## Abstract

The greater amberjack (*Seriola dumerili*) is an emerging marine fish that is increasingly favored in aquaculture. Currently, there are few studies on the development and regulation of greater amberjack ovaries. In this study, the ovary transcriptome profiles of greater amberjack at three different stages (stage II, III, and IV) were performed, and identified the genes and pathways that may play significant roles in the processes of follicle growth and maturation. A total of 6597, and 1061 differentially expressed genes (DEGs) were detected in FII vs. FIII, FIII vs. FIV, and FII vs. FIV stages, respectively. GO and KEGG enrichment analyses revealed that these DEG_S_ are primarily involved in steroid hormone biosynthesis (e.g., *cyp11a1*, *cyp17a1*, *cyp19a1a*, *hsd3b1*, *esr1*), lipid metabolism (e.g., *plpp3*, *lpl*, *pld1,* and *fabp10a*), and meiotic arrest and resumption (e.g., *pgr*, *arb*, *ccnd2*, *adcy2*, *adcy9*, *myl9*, *calm1*). Additionally, several signaling pathways involved in ovarian development have been identified, including the PI3K-Akt, Wnt, TGF-beta, GnRH, and immune-related signaling pathways. qPCR results of nine representative genes related to steroid hormone synthesis and cell growth verified the reliability of the generated RNA-seq data. This research contributes to our comprehension of the molecular processes underlying ovarian growth and maturation in marine fishes and provides a theoretical basis for the investigation of functional genes associated with oogenesis in greater amberjack.

## Simple Summary

The greater amberjack (*Seriola dumerili*) is an emerging marine fish species that has become a sought-after aquaculture product due to its tender flesh and rapid growth rate. Ovarian development is closely related to the production of high-quality eggs and serves as a critical determinant of aquaculture yield. However, studies on ovarian development in greater amberjack remains limited. This study aims to screen and identify candidate genes and potential signaling pathways involved in greater amberjack ovarian development through transcriptome analysis, thereby providing a foundation for further elucidation of the molecular mechanisms underlying ovarian maturation in fish.

## 1. Introduction

In recent years, the demand for aquaculture species, particularly fish, has increased significantly due to overfishing, which has resulted in a decline in natural resources. Producing large numbers of healthy and viable eggs with high survival rates is pivotal to the success of aquaculture [1]. The ovary serves as the site of oogenesis [2], and ovarian development is closely related to the production of high-quality eggs. The significant developmental events occurring in the ovaries of teleosts are similar, involving the proliferation of oogonia and the formation of primary oocytes that subsequently grow within the follicles, form cortical alveoli, enter vitellogenesis, undergo maturation, and are ultimately ovulated as fertilized eggs [3]. Oocyte growth, a vital stage of oogenesis, has been extensively investigated across various species. Fish follicle development can be divided into two main phases: the primary growth phase (PG) and the secondary growth phase (SG) [4]. The SG can further be subdivided into the following stages: primary vitellogenic oocytes (PV), vitellogenic oocytes (early vitellogenic oocytes (EV) and middle vitellogenic oocytes (MV)), fully grown oocytes (FG), and mature oocytes (M) [5]. PG encompasses the developmental stage of oocytes from the meiotic chromatin–nucleolus stage to the early cortical alveoli (CA) stage. The onset of SG is characterized by the emergence and accumulation of CA, a phase referred to as “primary vitellogenesis” [4]. True vitellogenesis involves the formation of yolk granules at the periphery of the cytoplasm as the oocyte matures [1,6]. By the conclusion of this process, the oocyte is enriched with maternal mRNAs, lipids, proteins, vitamins, carbohydrates, and hormones essential for subsequent maturation and early embryonic development [1]. Subsequently, the FG oocytes experience meiotic maturation, which involves the resumption of meiosis and the finalization of the first meiotic division following growth completion and culminating in the formation of fertilizable eggs [1].

Ovarian development is a complex and dynamic physiological process in fish reproduction, which is influenced by various factors, including the stimulation of environmental factors, the accumulation of nutrients, the regulation of steroid hormones, and genetic factors [7,8]. In yellowtail (*Seriola quinqueradiata*), comprehensive regulation of water temperature and photoperiod may enhance the levels of follicle-stimulating hormone (FSHβ) and luteinising hormone (LHβ), leading to increased serum estradiol (E_2_) and testosterone (T) levels, which ultimately facilitate maturation [9]. Furthermore, in yellowfin sea bream (*Acanthopagrus latus*) and flame angelfish (*Centropyge loriculus*), the relative fecundity, hatching rate, and larval survival rate were significantly improved by the supplementation of higher levels of n-3 long-chain polyunsaturated fatty acids (LC-PUFA) in their diet [10,11]. Genetic factors are considered to be among the most critical factors. With the rapid advancement of high-throughput sequencing technology, RNA–Seq has emerged as a crucial tool for studying fish gonad advancement, primarily used for screening relevant candidate genes and signaling pathways [12,13,14]. Jiang et al. analyzed the transcriptomes of stage III and IV ovaries of the spotted scat (*Scatophagus argus*) and found several differentially expressed genes (DEGs) associated with ovarian maturation, including *fshr*, *cyp17a1*, *foxl2*, *ppara*, *fabp3*, etc., which are highly expressed in stage IV [15]. In addition, Jia et al. compared the ovaries of juvenile and adult Yellow River carp (*Cyprinus carpio*) and found that genes involved in oocyte meiosis, maturation, DNA modification, and fertilization were significantly up-regulated in adults [2]. Li et al. performed transcriptome analysis on the stage I and stage III ovaries of golden pompano (*Trachinotus ovatus*) and found that *gnrhr*, *fshr*, *fshβ*, *cyp11a*, *sirt3*, and *peg3* were highly expressed in stage III ovaries [16]. Furthermore, a comparative transcriptome analysis of wild and hatchery-produced greater amberjack at maturity revealed the up-regulation of factors associated with hypogonadism and polycystic ovary syndrome in mammals [17,18]. In summary, most previous studies on fish ovaries have primarily focused on the early and mature stages, with comparatively fewer investigations into the intermediate processes. A comprehensive understanding of the molecular mechanisms that govern these distinct stages of ovarian development is essential for advancing our knowledge of fish reproduction.

The greater amberjack (*Seriola dumerili*) is a marine, large migratory, pelagic fish with a circumglobally distribution in tropical and temperate waters [19]. This species holds significant value for commercial and recreational fisheries as well as aquaculture due to its rapid growth rate, considerable body size, high-quality flesh, and high worldwide consumer acceptability. Field sampling indicates that a minority of females reached sexual maturity at 1 year of age, 62.7% reached sexual maturity by 2 years, 90.4% reached sexual maturity by 3 years, and 100% reached sexual maturity by 6 years of age and beyond [20,21]. The reproductive potential of the greater amberjack is enormous and artificial induction of spawning has been successfully achieved in some laboratories using gonadotropin-releasing hormone agonists (GnRHa), which are administered via injection or sustained release implants, as well as human chorionic gonadotropin (hGC) injections administered during the advanced phase of spermatogenesis and just prior to the expected spawning season [22,23,24,25,26,27]. However, the efficiency of artificial reproduction remains low in practical breeding scenarios, primarily due to the scarcity of female fish capable of producing a large quantity of healthy and viable eggs [28]. A deeper understanding of ovarian development could provide a theoretical foundation for enhancing the quality and quantity of eggs produced by the female greater amberjack. Currently, the differential expression of miRNAs in the three different ovarian development stages (immature, maturing (late vitellogenesis), and spent ovaries) was examined, and several miRNAs associated with follicular atresia, apoptosis, and cell death were identified [29]. To further address some gaps in the existing literature in this area, the present study analyzes the transcriptomes of the ovary at the PG stage (stage II), PV stage (stage III), and MV stage (stage IV). This study helps to screen and identify candidate genes and potential signaling pathways involved in ovarian development in greater amberjack, thereby providing a basis for further elucidating the molecular mechanism of ovarian maturation in fish.

## 2. Materials and Methods

### 2.1. Experimental Fish and Sample Collection

Twelve two-year-old female greater amberjack (body weight, 3.60–4.25 kg; total length, 64–72 cm) were sampled from Fuminyang Aquaculture Development Co., (Zhangzhou, China) between April and June 2023 (Table S1). Prior to sample collection, tricaine methane sulfonate (MS-222, Sigma, Saint Louis, MO, USA) (100 mg/L) was utilized to anesthetize the experimental fish. The ovarian tissues were rapidly isolated and weighed. These tissues were then divided into two portions: one portion was placed in RNA Later solution (Beyotime, Shanghai, China) overnight at 4 °C and subsequently transferred to a −80 °C refrigerator for total RNA extraction. The other portion was fixed in Bouin’s solution (Phygene, Fuzhou, China) for 48 h and subsequently characterized histologically using hematoxylin–eosin staining (HE). The gonadosomatic index (GSI) was calculated using the formula GSI  =  (gonad weigh)/(body weight).

### 2.2. Histological Observation of Ovaries

Dissected greater amberjack ovaries were immersed in Bouin’s solution for 48 h at room temperature for fixation. Following fixation, the ovarian tissues were dehydrated in ethanol, cleared in xylene, and embedded in paraffin. Ovarian blocks were then sectioned (width, 5–7 μm) using a rotary microtome (HistoCore BIOCUT, Leica). Hematoxylin-eosin (HE) staining was performed as our previous study described [30], and images of the stained tissue sections were captured using a color microscope camera (DFC7000 T, Leica, Wetzlar, Germany). The different developmental stages (PG stage (stage II), PV stage (stage III), and MV stage (stage IV)) of the ovaries in the experimental fish were determined based on our previous study [31].

### 2.3. RNA Extraction, Library Preparation, and Sequencing

Based on the three ovarian developmental stages (PG stage (stage II), PV stage (stage III), and MV stage (stage IV)) observed in the tissue sections (three samples were taken from each stage), ovarian tissues from nine out of 12 fish were selected for total RNA extraction using Trizol (Invitrogen, Carlsbad, CA, USA) according to the manufacturer’s instructions. A total of 1.5% agarose gel electrophoresis was employed to evaluate RNA integrity, while RNA concentration and quality were assessed using a Nanodrop spectrophotometer (Thermo Scientific, Wilmington, DE, USA). Each sample exhibited an RNA concentration exceeding 200 ng/μL, with a total amount greater than 10 μg. The NEBNext^®^ UltraTM RNA Library Prep Kit was utilized to construct complementary DNA (cDNA) libraries. Briefly, ovary messenger RNA (mRNA) with a poly A tail was enriched from total RNA using Oligo (dT) magnetic beads and subsequently fragmented randomly using a fragmentation buffer. Employing random oligonucleotides as primers, fragmented mRNA was then reverse transcribed into cDNA using the NEBNext Ultra RNA Library Prep Kit from Illumina (New England Biolabs, Ipswich, MA, USA). The synthesized double-stranded cDNA was purified using AMPure XP beads (1.0), followed by end repair, adaptor ligation, fragment sorting, and size selection. Polymerase chain reaction (PCR) amplification was used to generate cDNA libraries, which were sequenced on the Illumina Novaseq 6000 from Gene Denovo Biotechnology Co. (Guangzhou, China).

### 2.4. Sequence Assembly, Annotation, and Functional Analysis

The high-quality clean reads were obtained by removing those containing adapters, exceeding 10% of unknown nucleotides (N) and low-quality reads from the original sequence via fastp (version 0.18.0). Meanwhile, the Q20, Q30, and GC contents of the clean reads were calculated. Next, by using HISAT (version 2.2.4) the high-quality clean reads were aligned with the reference genome of greater amberjack (Accession numbers: BDQW01000001-BDQW01034655, Biosample ID: SAMD00083043) [32]. The greater amberjack genome assembly measures 678 Mbp in length, with an N50 of 5.8 Mbp. Clean RNA-seq data were assembled using StringTie (version 1.3.1) with default parameters. Gene expression levels were calculated and normalized to the fragments per kilobase of transcript per million mapped reads (FPKM) value. To investigate the dynamic changes of different ovarian stages in greater amberjack, three comparison groups were constructed, including FII vs. FIII, FII vs. FIV, and FIII vs. FIV. Principal component analysis (PCA) analysis was performed on the FPKM scores to assess differences between and within groups. Differential expression analysis was performed using DESeq2 software (version 1.20.0), and differentially expressed genes (DEGs) were identified using |log_2_(fold change)| > 1 and a false discovery rate (FDR) < 0.05 as a threshold. Then, Gene Ontology (GO) and Kyoto Encyclopedia of Genes and Genomes (KEGG) pathway databases enrichment analysis was carried out to identify significant DEGs. The genes associated with the GO terms and the KEGG pathway were identified. And the pathways with a *p*-value < 0.05 were considered significant.

### 2.5. Real-Time Quantitative PCR (qRT-PCR) Validation of DEGs

To confirm the reliability of the transcriptome sequencing results, qRT-PCR validation was conducted on a random selection of 22 differentially expressed genes (DEGs). The samples used for qRT-PCR and RNA-seq were from the same batch. cDNA synthesis was performed through reverse transcription using the TransScript^®^ One-Step gDNA Removal and cDNA Synthesis SuperMix (TransGen Biotech, Beijing, China) according to the manufacturer’s instructions. Gene-specific primer sequences were designed using Primer 6 software (Premier Biosoft International, Palo Alto, CA, USA) based on greater amberjack transcriptome data, with *β-actin* as the internal reference gene [33,34,35]. The primer sequences are listed in Table S2.

The qRT-PCR was performed employing the qPCR PerfectStart TM Green qPCR SuperMix (TransGen Biotech, Beijing, China) on a Light Cycler 96 (Roche, Indianapolis, USA). The PCR reaction mixture, totaling 20 μL, contained 10 μL of 2 × qPCR SuperMix, 6.2 μL of RNase-free water, 2 μL of cDNA, and 0.8 μL of both forward and reverse primers (10 μmol/mL). The thermal cycling protocol was set as follows: initial denaturation at 95 °C for 5 min, succeeded by 40 cycles of denaturation at 95 °C for 30 s, annealing at 60 °C for 20 s, and extension at 72 °C for 20 s. Additional amplification efficiencies and correlation coefficients (R^2^) of each primer were determined by standard curves for fivefold dilutions (1/5, 1/25, 1/125, 1/625, and 1/3125) of the cDNA template. The relative gene expression levels were determined using the comparative threshold cycle method, calculated as R = 2^−ΔΔCt^.

### 2.6. Statistical Analysis

The expression levels of DEGs and their FPKM values are presented as mean ± standard error (SE). Statistical analysis was conducted using IBM SPSS Statistics 22.0 software. One-way analysis of variance (ANOVA) with Duncan’s post-hoc test was used to detect statistically significant differences among groups. A significance threshold was established at *p* < 0.05.

## 3. Results

### 3.1. Classification of Ovarian Developmental Stages II, III, and IV

**Figure 1:**
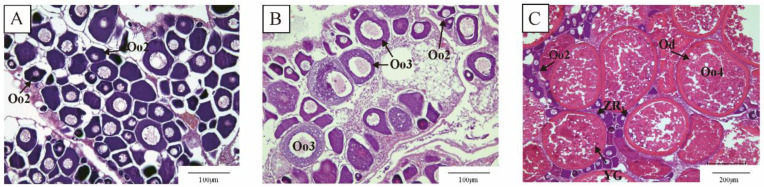
Ovarian histology of greater amberjack. (**A**) stage II. (**B**) stage III. (**C**) stage IV. Oo2: primary growth stage oocytes (stage II); Oo3: primary vitellogenic-stage oocytes (stage III); Oo4: middle vitellogenic-stage oocytes (stage IV); Yg: yolk granules; Od: oil droplets; ZR: zona radiata.

Based on the histological characteristics of the greater amberjack ovary (Figure 1), three representative stages of ovarian development were identified: stage II, stage III, and stage IV. In stage II, the ovary primarily consisted of small, predominantly circular or irregular-shaped oocytes at stage II in the growth phase, exhibiting a large number of nucleoli of varying sizes. The yolk nucleus was distributed in a circular arrangement around the nucleus within the cytoplasm, and a single layer of follicular membrane was present at the periphery of the oocyte (Figure 1A). In stage III, the ovaries consisted mainly of oocytes at the primary growth stage and those at the cortical vesicle stage. There was a significant increase in the volume of both oocytes and nuclei, with lipid droplets appearing in the cytoplasm (Figure 1B). In stage IV, the ovary was predominantly composed of preovulatory oocytes, which were significantly larger and filled with yolk granules and lipid droplets within the cytoplasm (Figure 1C). Additionally, the gonadosomatic index (GSI) increased with ovarian maturation (Figure S1).

### 3.2. RNA-Seq Data Statistics

The Illumina Novaseq 6000 system was used to sequence the nine ovary samples from three different developmental stages. A total of 434,000,339 raw reads were obtained from the greater amberjack ovary transcriptome, including 143.64 million in the stage II group, 157.77 million in the stage III group, and 132.58 million in the stage IV group. After adapter sequences and low-quality reads, 43,259,336 clean reads (99.68% of raw reads) were retained, including 143.19, 157.2, and 132.15 million in stage II, stage III, and stage IV, respectively. The percentages of Q20 and Q30 exceeded 97.53% and 93.02%, respectively. And the GC content was approximately 50%, suggesting that the sequencing outcomes were reliable. The clean reads from all nine ovary sequencing libraries were then mapped to the greater amberjack reference genome (Accession numbers: BDQW01000001-BDQW01034655, Biosample ID: SAMD0008304), with a total of 464.10 million reads successfully mapped, of which 339.80 million reads were uniquely mapped (Table 1).

### 3.3. DEGs Profiles in Stage II, III, and IV Ovaries

**Figure 2:**
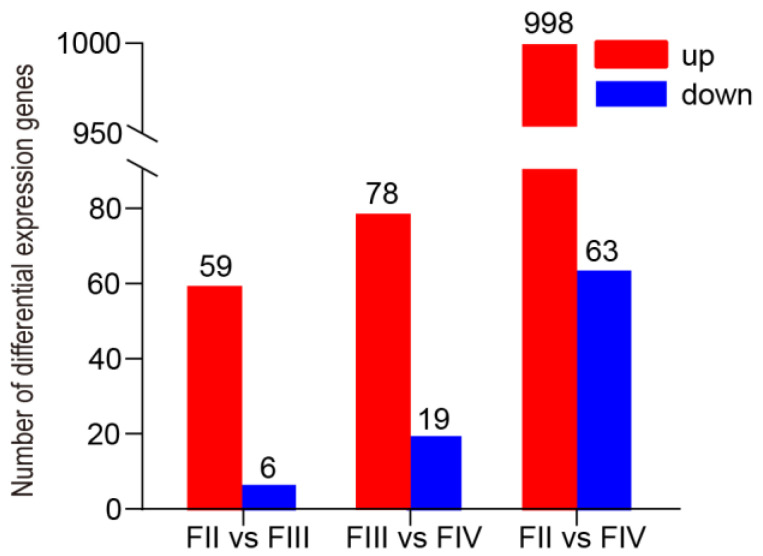
Differentially expressed genes (DEGs) profiles in greater amberjack ovaries at different developmental stages (FII, FIII, and FIV). The DEGs (|log_2_(FC)| ≥ 1 and FDR < 0.05) of FII vs. FIII group, FIII vs. FIV group, and FII vs. FIV.

Principal component analysis (PCA) of the transcriptome delivery samples revealed that the biological replicates of the groups clustered together, thereby demonstrating the feasibility of ovarian-stage grouping (Figure S2). Differential expression data from different stages of greater amberjack ovarian development are shown in Figure 2. The comparative expression profile between stage II and III showed 65 DEGs. Compared to the stage II group, 59 and six genes were up- and down-regulated in the stage IV group, respectively. Moreover, a total of 97 DEGs (78 up-regulated and 19 down-regulated) and 1061 (998 up-regulated and 63 down-regulated) DEGs were identified in the FIII vs. FIV and FII vs. FIV groups, respectively (Figure 2). Among these, the top 10 up- and down-regulated DEGs are displayed in Table S2. 

### 3.4. Functional Annotation and Pathway Analysis of DEGs

**Figure 3:**
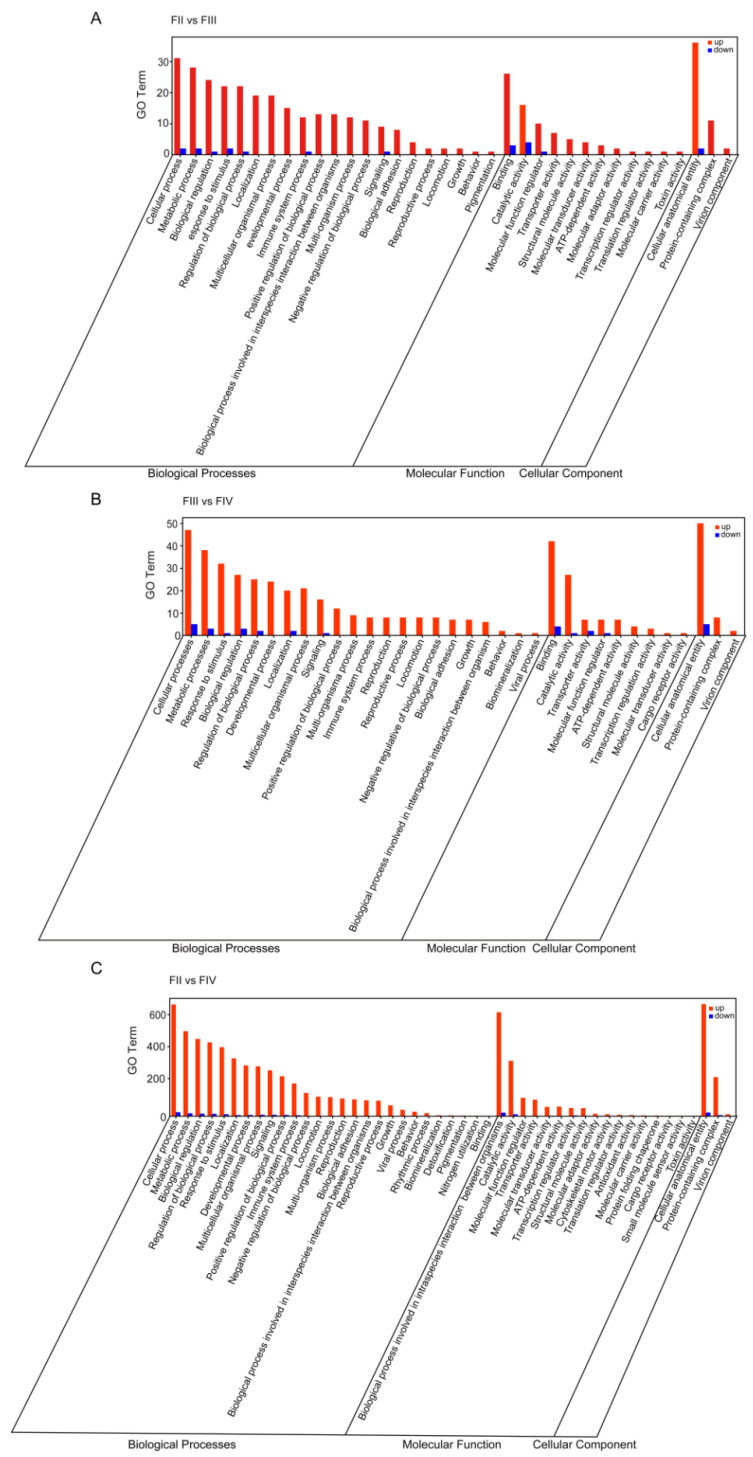
Gene Ontology (GO) enrichment analysis of ovarian DEGs at different developmental stages. (**A**) FII vs. FIII group, (**B**) FIII vs. FIV group, (**C**) FII vs. FIV group. The red and blue bars represent the number of up- and down-regulated DEGs, respectively; the X and Y-axis represent the enriched pathways and the number of DEGs, respectively.

The results of the GO enrichment analysis showed that the DEGs were classified into three main functional categories: Biological Process (BP), Molecular Function (MF), and Cellular Component (CC). The enrichment of GO category terms in BP, CC, and MF between three ovarian developmental stages are shown in Figure 3 and Table S3. In the FII vs. FIII, FIII vs. FIV, and FII vs. FV comparison groups, cellular process (GO:0009987), metabolic process (GO:0008152), response to stimulus (GO:0050896), biological regulation (GO:0065007), and regulation of biological process (GO:0050789) were the most enriched GO terms in the BP category. Binding (GO:0005488), catalytic activity (GO:0003824), transporter activity (GO:0005215), and molecular function regulator (GO:0098772) were highly ranked in the MF category. In the CC category, cellular anatomical entity (GO:0110165), protein-containing complex (GO:0032991), and virion component (GO:0044423) were markedly (Figure 3).

**Figure 4:**
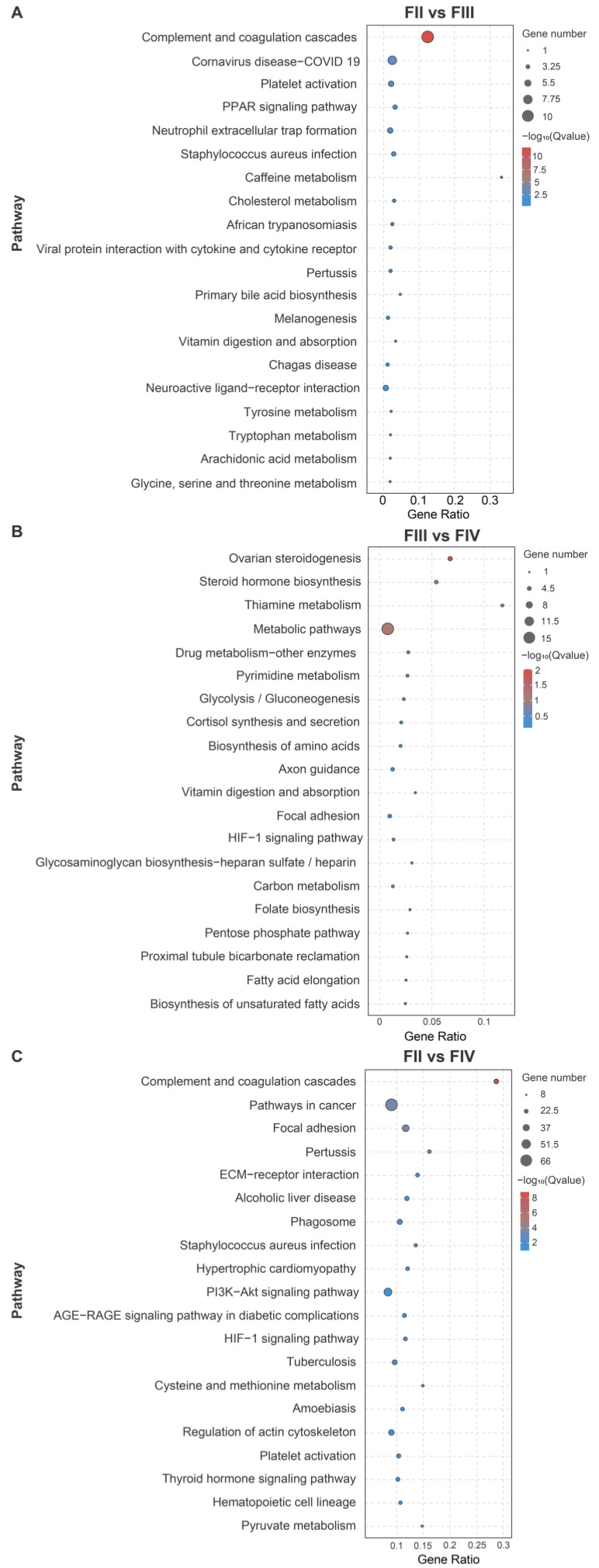
The most enriched KEGG pathway from greater amberjack ovarian DEGs at different developmental stages. (**A**) FII vs. FIII group, (**B**) FIII vs. FIV group, (**C**) FII vs. FIV group. The black circles represent the number of DEGs. The colors indicate the −log_10_(Q-value) of DEGs. The horizontal axis is the ratio of KEGG pathway annotated DEGs to the total number of DEGs.

KEGG pathway enrichment analysis showed that 11 pathways were significantly enriched (*p* < 0.05) in FII vs. FIII. The 11 pathways could be divided into four categories: environmental information processing (e.g., Viral protein interaction with cytokine and cytokine receptor), metabolism (e.g., Caffeine metabolism), organismal systems (e.g., complement and coagulation cascades, platelet activation, PPAR signaling pathway, neutrophil extracellular trap formation, and cholesterol metabolism), and human diseases (e.g., coronavirus disease-COVID-19 elt, (Figure 4A). For DEGs in FIII vs. FIV, metabolism pathways (steroid hormone biosynthesis, thiamine metabolism, metabolic pathways, drug metabolism-other enzymes, pyrimidine metabolism, and glycolysis/gluconeogenesis) and ovarian steroidogenesis were significantly enriched (Figure 4B). Similar to FII vs. FIII, 54 pathways were found to be significantly enriched (*p* < 0.05) in the FII vs. FIV and could also be divided into the five categories (Figure 4C). Among these pathways, complement and coagulation cascades, pathways in cancer, focal adhesion, and pertussis pathways were the most predominant. The significant KEGG enrichment pathways are listed in Table S5.

### 3.5. Validation by qRT-PCR

**Figure 5:**
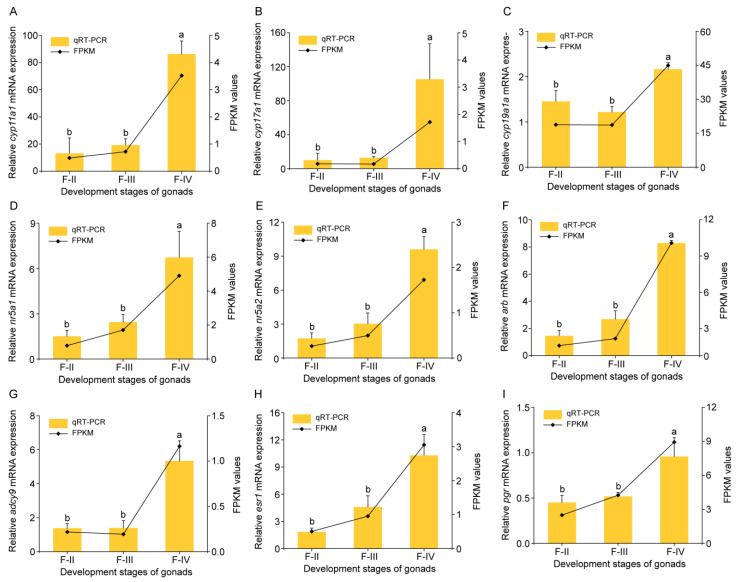
Validation of DEGs related to ovarian development by qRT–PCR. The relative level of mRNA transcripts was measured using qRT-PCR via the 2^−∆∆Ct^ method. Data are expressed as means ± standard error (SE) (*n* = 3). The reference gene used was *β-actin*. Different lowercase letters above the error bars indicate statistical differences at *p* < 0.05 between different development stages, as assessed by one-way analysis of variance (ANOVA) followed by Duncan’s post hoc test. (**A**) Relative expression level of *cyp11a1*; (**B**) Relative expression level of *cyp17a1*; (**C**) Relative expression level of *cyp19a1a*; (**D**) Relative expression level of *nr5a1*; (**E**) Relative expression level of *nr5a2*; (**F**) Relative expression level of *ar*; (**G**) Relative expression level of *adcy9*; (**H**) Relative expression level of *esr1*; (**I**) Relative expression level of *pgr*.

To validate the RNA-seq data, 14 DEGs were randomly selected between three different ovarian developmental stages (II, III, and IV) and quantitatively analyzed by qRT-PCR. The findings revealed that the trends in gene expression among the three stage groups were consistent with the observations from the RNA-seq data, validating the accuracy of the RNA-seq analysis (Figure S3). We also examined 10 DEGs involved in steroid hormone synthesis and cell growth based on the RNA-seq data, including *cyp11a1*, *cyp17a1*, *cyp19a1a*, *nr5a1*, *nr5a2*, *arb*, *adcy9*, *esr1*, and *pgr*. These genes exhibited high expression levels in mature oocytes (Figure 5).

To validate the RNA-seq data, 14 DEGs were randomly selected between three different ovarian developmental stages (II, III, and IV) and quantitatively analyzed by qRT-PCR. The findings revealed that the trends in gene expression among the three stage groups were consistent with the observations from the RNA-seq data, validating the accuracy of the RNA-seq analysis (Figure S3). We also examined 10 DEGs involved in steroid hormone synthesis and cell growth based on the RNA-seq data, including *cyp11a1*, *cyp17a1*, *cyp19a1a*, *nr5a1*, *nr5a2*, *arb*, *adcy9*, *esr1*, and *pgr*. These genes exhibited high expression levels in mature oocytes (Figure 5).

### 3.6. Expression of Representative DEGs in Ovarian Growth and Development

#### 3.6.1. Expression of DEGs Associated with Steroid Hormone Biosynthesis

**Figure 6:**
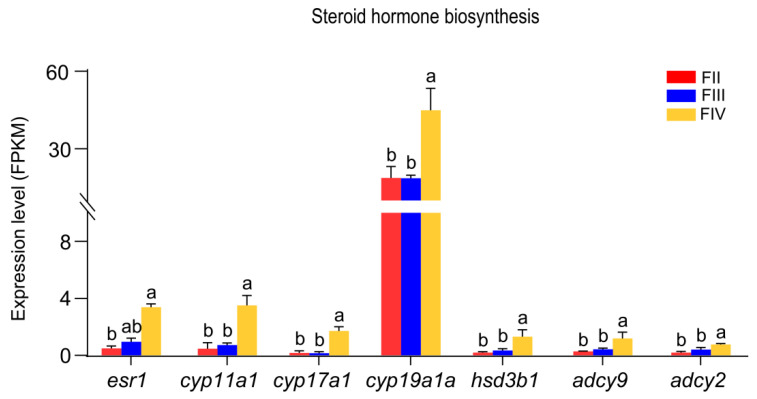
Bar graphs showing the expression of DEGs associated with steroid hormone synthesis in stage II, III, and IV ovary of greater amberjack. These data are based on the FPKM values obtained from the RNA-seq data. Data are expressed as means ± standard error (SE) (*n* = 3). Different lowercase letters above the error bars indicate statistical differences at *p* < 0.05 between different development stages, as assessed by one-way analysis of variance (ANOVA) followed by Duncan’s post hoc test.

Seven DEGs involved in steroid hormone biosynthesis were identified from the RNA-seq data. No significant changes in the expression of these seven genes were observed between stages II and III. However, six genes (*cyp11a1*, *cyp17a1*, *cyp19a1a*, *hsd3b*, *adcy2,* and *adcy9*) were significantly up-regulated from stage III to stage IV, while the *esr1* expression was not significantly different. From stage II to stage IV, the expression of these seven genes was significantly up-regulated (Figure 6).

#### 3.6.2. Expression of DEGs Associated with Lipid Metabolism

**Figure 7:**
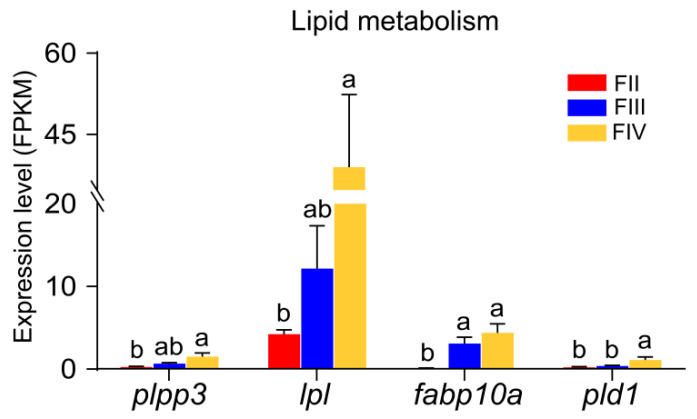
Bar graphs showing the expression of DEGs involved in lipid metabolism in stage II, III, and IV ovary of greater amberjack. Different lowercase letters above the error bars indicate statistical differences at *p* < 0.05 between different development stages.

The accumulation of lipids is an important aspect of follicle development. Based on the KEGG pathway enrichment results, we identified four genes that may be associated with lipid metabolism. From stage II to stage III, only the *fabp10a* expression level was significantly up-regulated, while other genes (*plpp3*, *lpl*, *pld1*) showed no significant changes. From stage III to stage IV, only *pld1* showed significant up-regulation, while other genes (*plpp3*, *lpl*, *fabp10a*) showed no significant difference (Figure 7). In addition, all genes were significantly up-regulated in stage IV compared to stage II.

#### 3.6.3. Expression of DEGs Associated with Meiotic Arrest and Resumption

**Figure 8:**
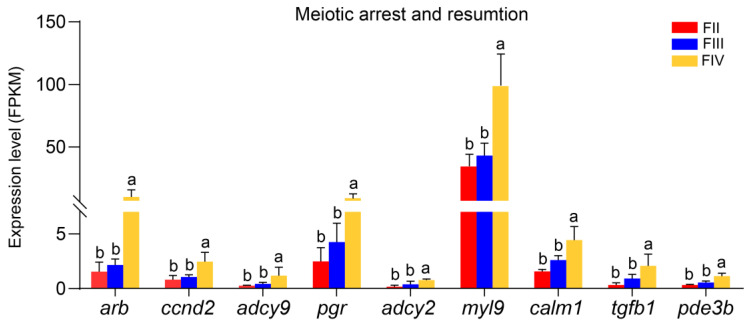
Bar graphs showing the expression levels of DEGs involved in meiotic arrest and resumption in stage II, III, and IV ovary of greater amberjack. Different lowercase letters above the error bars indicate statistical differences at *p* < 0.05 between different development stages.

The transcriptomic data identified nine DEGs (*arb*, *ccnd2*, *adcy9*, *pgr*, *adcy2*, *myl9*, *calm1*, *tgfb1,* and *pde3b*) involved in meiotic arrest and resumption. These genes were classified into two categories: genes associated with cell cycle and meiosis and those related to cAMP or cGMP signaling pathway. There is no significant difference in the expression of these nine genes between stage II and stage III. However, their expression levels showed significant up-regulation in stage IV compared to stage II and III (Figure 8).

#### 3.6.4. Expression of DEGs Associated with Ovarian Developmental Pathways

**Figure 9:**
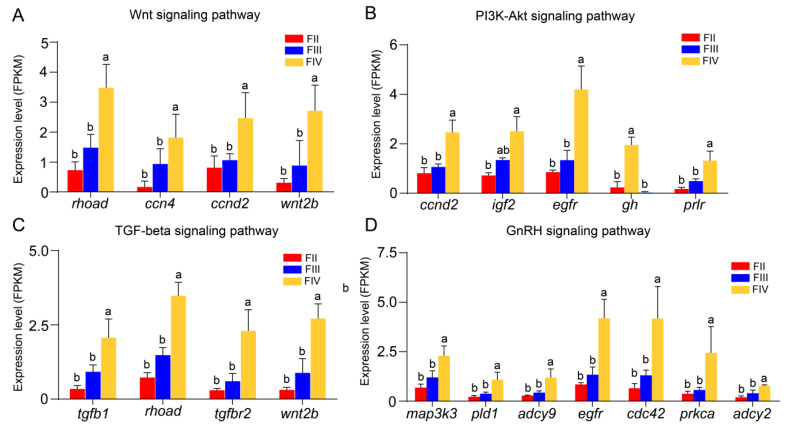
Bar graphs showing the expression levels of DEGs in signaling pathways associated with follicle development in stage II, III, and ovary of greater amberjack. Different lowercase letters above the error bars indicate statistical differences at *p* < 0.05 between different development stages.

In this study, some key signaling pathways related to ovarian growth and development were identified from the transcriptome data, such as PI3K-Art (*ccnd2*, *igf2*, *egfr*, *gh*, and *prlr*), Wnt (*rhoad*, *ccn4*, *ccnd2*, and *wnt2b*), TGF-beta (*tgfb1*, *rhoad*, *thfbr2*, and *wnt2b*), and GnRH signaling pathway (*map3k3*, *pld1*, *adcy9*, *cdc42*, *prkca*, *adcy2*). Among these DEGs, the expression levels of *ccnd2*, *egfr*, *prlr*, *rhoad*, *ccn4*, *wnt2b*, *tgfb1*, *thfbr2*, *map3k3*, *pld1*, *adcy9*, *cdc42*, *prkca*, and *adcy2* did not exhibit significant differences between stage II and III. However, their expression levels were significantly up-regulated in stage IV compared to both stage II and III. Additionally, the expression level of *gh* was significantly higher in stage II ovaries than in stage III and IV. Furthermore, the *igf2* mRNA levels in stage IV ovaries was significantly elevated compared to stage II and III (Figure 9).

## 4. Discussion

High-quality eggs are essential for the successful artificial reproduction of fish in the aquaculture industry. Fish ovarian development has been extensively studied, providing a theoretical basis for promoting the formation of mature eggs. In this study, we identified DEGs in the ovaries at different stages of 2-year-old greater amberjack through RNA-seq. This methodology facilitated the identification of genes and pathways involved in the molecular regulation of steroid hormone synthesis, lipid droplet and yolk production, and meiosis in greater amberjack. The findings of this research enhance our understanding of ovarian development regulation, which may be beneficial for artificial reproduction in this economically important species.

### 4.1. The DEGs Involved in Steroid Hormone Synthesis

In this study, several DEGs associated with the steroid hormone biosynthesis in greater amberjack were screened via the KEGG pathway, which are involved in the regulation of ovarian development and maturation. The biosynthesis of steroid hormones is facilitated by enzymes from two primary classes: cytochrome P450 monooxygenases (CYPs) and hydroxysteroid dehydrogenases (HSDs) [36,37].

CYP11A1, CYP17A1, and CYP19A1A are three cytochrome P450 hydroxylases that catalyze the successive steps of steroidogenesis in vertebrates [36,38]. The cholesterol side-chain cleavage enzyme encoded by *cyp11a1* facilitates the synthesis of pregnenolone, which serves as a precursor for all steroid hormones and is essential for steroid hormone synthesis in vertebrates. This enzyme also promotes follicular development and oocyte maturation to some extent [39,40,41]. In black rock (*Sebastes schlegelii*), the expression of *cyp11a1* increases with gonadal development and is highly expressed in mature ovaries [41], aligning with our research findings. 17 α-hydroxylase/17,20-lyase (Cyp17 or P450c17) plays a crucial role in controlling steroid synthesis and is considered one of the key rate-limiting enzymes in the steroid hormone biosynthetic pathway [42,43,44]. Teleosts typically possess two Cyp17 subtypes, Cyp17a1 and Cyp17a2. Cyp17a1 exhibits both hydroxylase and lyase activities, whereas Cyp17a2 is limited to hydroxylase activity [45,46]. In rainbow trout and Japanese eel, *cyp17a1* gene expression is significantly elevated during the mature stage of ovarian development [47,48]. Additionally, in spotted scat and greater amberjack ovaries, *cyp17a1* expression levels show a progressive increase from stage II to stage IV [49]. Aromatases cytochrome (P450 arom/CYP19), also known as estrogen synthetase, catalyzes the conversion of androgen into estrogen, which are crucial for gonadal differentiation and sexual maturation [50,51,52]. In most teleosts, two types of *cyp19* genes have been identified in the gonad and brain: *cyp19a1a* and *cyp19a1b*. The *cyp19a1a* gene encodes the gonad-specific aromatase, or ovarian aromatase, while *cyp19a1b* encodes the brain-specific aromatase, or neural aromatase [53,54]. In the orange-spotted grouper (*Epinephelus coioides*), mRNA expression of *cyp19a1a* was significantly enhanced at stages IV and V, showing a trend consistent with the changes in estradiol (E_2_) content [55]. Similar results were also observed in Japanese flounder and greater amberjack, where *cyp19a1a* expression significantly increased from the ovary at stage II to stage IV [56]. These findings indicate the essential role of *cyp19a1a* in estrogen synthesis, which regulates ovarian development, maturation, and female reproduction in teleosts. Notably, *foxl2*, *nr5a1,* and *nr5a2* are considered upstream regulators of *cyp19a1a* [57,58]. In this experiment, the expression of three upstream genes exhibited a similar trend of *cyp19a1a*, with a gradual increase in expression (Table S6). This increase may facilitate the full expression of *cyp19a1a*, thereby influencing estradiol production and contributing to follicle development and maturation in the ovaries of greater amberjack.

The *hsd3b* gene encodes 3β-hydroxysteroi dehydrogenase/Δ(5)-Δ(4)-isomerase, which catalyzes the synthesis of δ4 steroids in the adrenals and gonads, a necessary step in the biosynthesis of sex hormones, glucocorticoids, and mineralocorticoids [59,60]. In adult medaka, strong *hsd3b* mRNA signals are typically detected in the granulosa cells of vitellogenic ovarian follicles, with *hsd3b* expression patterns correlating with the capacity for estrogen production during folliculogenesis [61]. In greater amberjack, the expression level of *hsd3b1* in stage IV ovaries is significantly higher than in stages II and III. Estrogen, a vital steroid hormone in the reproductive process of vertebrates, primarily signals through specific intracellular estrogen receptors (ERs), including *esr1* (encoding ERα), *esr2a, and esr2b* (encoding ERβ1 and ERβ2, respectively). The highest levels of *esr1* expression are observed in the ovary and liver tissues of yellow perch (*Perca flavescens*) [62]. Furthermore, in medaka and Korean rockfish (*Sebastes schlegelii*), high levels of *esr1*, *esr2a,* and *esr2b* expression are observed during ovarian development [63,64]. In greater amberjack, there is no significant difference in the expression levels of *esr2a* and *esr2b* among the three stages of ovarian development (Table S6), while the *esr1* expression levels are significantly higher in stage IV than in stage II. This suggests that the ovarian development process in greater amberjack may be more closely associated with *esr1*. The findings of the current study indicate that the expression levels of genes involved in steroid hormone biosynthesis increase as the ovary matures, peaking in stage IV ovaries. This finding suggests that elevated hormone levels are essential for maintaining ovarian function during maturation, thereby confirming the critical role of steroid hormones in ovarian physiology.

### 4.2. The DEGs Involved in Lipid Metabolism

Different lipid droplets, also referred to as cortical granules, appear in the cytoplasm, followed by the formation and accumulation of yolk granules [65] In Japanese flounder, transcriptome sequencing has revealed that numerous genes related to ovarian lipid metabolism are up-regulated from the primary growth ovarian stage to the secondary oocyte growth stage [65]. The gene *lpl* encodes lipoprotein lipase (LPL), which hydrolyzes triglycerides carried by chylomicrons and very low-density lipoproteins (VLDLs) into glycerol and fatty acids for storage and utilization by various tissues [66]. Lpl is predominantly expressed in tissues with a high demand for lipid oxidation or storage. In European seabass (*Dicentrarchus labrax* L.), *lpl* mRNA expression is localized to the follicular cells surrounding the oocyte, and higher *lpl* levels are observed in fish with increased ovarian maturity. This suggests that *lpl* is essential for the formation and accumulation of lipospheres in oocytes [67]. Herein, in stage IV ovaries, oocytes are filled with a large number of lipid droplets, and the expression of *lpl* is significantly elevated, indicating that *lpl* plays a conserved role in lipid absorption in greater amberjack oocytes. The fatty acid binding protein (*fabp*) super-family influences lipid and energy metabolism, as well as inflammatory responses, by regulating fatty acids and important intracellular signaling pathways [68]. The rainbow trout *fabp10* gene has been implicated in the regulation of host cell lipid deposition and liver metabolism [69]. Our findings of significant up-regulation of *fabp10* in greater amberjack stage III and IV ovaries further underscore its important role in ovarian lipid synthesis. Phospholipid phosphatase 3, encoded by *plpp3*, is a key regulator of lipid homeostasis, converting phosphatidic acid to diacylglycerol [70]. Phospholipase D (PLD) is an enzyme that plays a central role in metabolic processes and signaling pathways involving phospholipids [71]. In greater amberjack, the *plpp3* and *pld1* expression levels were gradually increased from stage II to IV ovaries, indicating their role in regulating ovarian phospholipids homeostasis.

### 4.3. The DEGs Involved in Meiotic Arrest and Resumption

In addition to the accumulation of nutrients and structural proteins in oocytes, many genes associated with meiosis may increase their expression prior to the conclusion of yolk production, ultimately facilitating ovarian maturation (meiosis resumption) [1,38]. In greater amberjack, the genes *arb*, *adcy9*, *pgr*, *pde3b*, *and mylf9* were predominantly enriched in the oocyte meiosis pathway. In giant freshwater prawn (*Macrobrachium rosenbergii*), estrogen-related receptor (*ERR*) may regulate ovarian development by influencing the expression of adenylate cyclase type 9 (*adcy9*) [72]. In the ovaries of ricefield eel and spotted scat, the *adcy* genes exhibited peak expression during the middle vitellogenic (MV) and late vitellogenic (LV) stages [15,38]. The androgen receptor (AR) has been identified as crucial for the development of follicular granulosa cells [73]. In primates, the expression of *arb* is positively correlated with granulosa cell proliferation and follicular growth, while being negatively correlated with granulosa cell apoptosis and follicular atresia, indicating that *ar* gene expression serves as a marker for healthy follicular growth [73]. Furthermore, in situ hybridization (ISH) results demonstrated that AR-positive signals were detected in the cytoplasm of all oocytes and in follicular cells of stage IV oocytes during the oogenesis process of large yellow croaker (*Larimichthys crocea*) [74]. In the current study, the *adcy9* and *arb* gene have the highest levels observed in stage IV ovaries, suggesting their important role in promoting follicular growth in greater amberjack. The progesterone receptor (PGR) is a nuclear receptor primarily regulated by its specific ligand, progesterone [75]. It plays a crucial role in the regulation of gonadal differentiation, gamete maturation, female ovulation, and male ejaculation in teleosts [76]. In zebrafish, homozygous knockout of *pgr* results in the failure of follicular cell rupture in mature follicles, leading to ovulation failure and female infertility [77]. In spotted scat, *pgr* is expressed in the early ovaries and gradually increases, reaching its maximum expression level at sexual maturity [76], which is consistent with our findings. Additionally, phosphodiesterase 3b (*pde3b*) was found to be enriched in the progesterone-mediated oocyte maturation signaling pathway. In mammals, increased oocyte PDE3 activity is essential for hydrolyzing cAMP to restore meiosis [78]. Furthermore, PDE observed in the mature stage of paddy eel oocyte ovaries may play a significantly role in the process of meiotic arrest and recovery [38]. Additional, in pigeons (*Columba livia*), *myl3*, *myl6*, and *myl9* are strongly associated with the promotion of preovulatory follicular maturation and ovulation [79]. Notably, many genes (*arb*, *adcy9*, *pgr*, *pde3b*, and *mylf9*) associated with the cell cycle, meiosis, and cAMP or cGMP synthesis and hydrolysis were up-regulated in stage IV ovaries compared to stage II and III, suggesting that oocyte meiosis was potentially restarted, leading to maturity.

### 4.4. Signaling Pathways Related to Oocyte Development

The mechanism of oocyte development involves many factors and cellular signaling pathways, including the Pl3K-Akt, the Wnt, the TGF-β, and the GnRH signaling pathways. Researches have shown that the PI3K/Akt pathway is associated with ovarian function, including primordial follicle recruitment, granulosa cell (GC) proliferation, corpus luteum formation, and oocyte maturation [80]. The GH/IGFs axis plays a significant role in influencing reproduction [81]. In the ovaries of mammals, growth hormone (*GH*) and insulin-like growth factor (*IGFs*) are crucial for promoting follicle growth, oocyte maturation, ovulation, and ovarian steroidogenesis [82,83,84,85]. In yellowtail (*Seriola quinqueradiata*), *igf1* and *igf2* enhance the expression of *cyp17a1* in the vitellogenic ovary via the PI3 kinase pathway [86]. In greater amberjack, ovarian *gh* expression significantly decreased from stage III to stage IV, while *igf2* exhibition gradually increased from stage II to IV, mirroring the expression pattern observed in zebrafish [81]. In contrast, *ghr* and *igf2r* are not expressed in the ovary, indicating that *gh* and *igf2* may indirectly influence ovarian development in greater amberjack by regulating other tissues external to the ovary. Members of the TGF-β superfamily have been identified as key regulating factors in the maturation of oocytes [81]. This superfamily is implicated in the regulation of follicular maturation in rainbow trout, potentially by influencing the synthesis and release of maturation-inducing hormone (MIH) [87]. In greater amberjack, the expression levels of *tgfb1* and *tgfbr2* were significantly elevated in stage IV compared to stage II and III, indicating their important role in the later stages of follicular development. WNTs are a class of signaling molecules that can effectively regulate the development of the female reproductive system. In mammals, WNT2 is expressed in granulosa cells throughout the entire follicular development process, promoting follicular granulosa cell proliferation via the WNT/CTNNB1 signaling pathway [88,89]. Notably, ovarian *wnt2* expression levels gradually increased from stage II to stage IV, indicating a potential association with granulosa cell proliferation and follicular development in greater amberjack ovaries. The gonadotropin-releasing hormone (GnRH) signaling pathway is critical in regulating the development and function of the reproductive system [90]. Furthermore, both the GnRH and the Wnt signaling pathway have been demonstrated to activate AP-1 (ERKs 1/2) within the MAPK pathway, which in turn stimulates pituitary gonadotropin secretion and facilitates follicle development [91,92]. In this study, most genes enriched in these pathways exhibited significant up-regulation from stage II to stage IV, further supporting the role of these pathways in follicular development.

The female reproductive organs have an indispensable immunity that guarantees the production of mature eggs of high quality. The innate and adaptive immune systems of the ovaries and fallopian tubes are critical in preventing pathogenic infections [93]. In greater amberjack, KEGG analysis indicated that the genes complement component 3 (*c3*), complement factor B (*cfb*) and fibrinogen gamma chain (*fgg*) were predominantly enriched in the complement and coagulation cascades, coronavirus disease-COVID-19, neutrophil extracellular trap formation, and *staphylococcus aureus* infection pathway. In Black rockfish (*Sebastes schlegelii*), both GO enrichment and KEGG analysis revealed that numerous DEGs from pre-mating (PRM)-vs-post-mating (POM) were associated with immune-related pathways, including antigen processing and presentation, immune response, and complement and coagulation cascade [94]. The functional annotation of ovarian fluid (OF) proteins in Siberian sturgeon (*Acipenser baeri*) highlighted their significant association with immune system processes, particularly the complement and coagulation cascade, neutrophil and leukocyte-mediated immunity [95]. This complex immune system in sturgeon OF may play vital roles in ovulation and in maintaining homeostasis following inflammation. Furthermore, an analysis of the top 20 KEGG pathways revealed significant enrichment in transcriptional misregulation in cancer, systemic lupus erythematosus, AGE-RAGE signaling pathway in diabetic complications, and MicroRNAs in cancer at various developmental stages of golden pompano ovaries [16]. These findings suggest that immune systems are crucial for ovarian development, and the stable physiological environment necessary for this development may be safeguarded against external pathogen threats through these immune mechanisms.

To sum up, the majority of the identified DEGs associated with ovarian development in greater amberjack did not exhibit significant differences between stage II (PG) and stage III (PV). This observation suggests that the changes in ovarian phenotype from stage II to III are not as pronounced as those observed from stage III to IV (MV). The secondary growth phase is characterized by nutrient accumulation. During this stage, numerous genes involved in steroid hormone biosynthesis (such as *cyp11a1*, *cyp17a1*, *cyp19a1a*, *hsd3b1*, and *esr1*), lipid metabolism (such as *plpp3*, *lpl*, *pld1*, and *fabp10a*), and meiotic arrest and recovery (such as *pgr*, *arb*, *ccnd2*, *adcy2*, *adcy9*, *myl9*, and *calm1*) are significantly upregulated in stage IV. This up-regulation stimulates the formation of lipid droplets and yolk granules, as well as the synthesis of sex steroid hormones, which are essential for subsequent maturation and early embryonic development.

## 5. Conclusions

In this study, the expression profiles in greater amberjack ovaries at different development stages (FII, FIII, and FIV stages) were analyzed by transcriptome. A total of 65, 97, and 1061 DEGs were identified in FII vs. FIII, FIII vs. FIV, and FII vs. FIV groups, respectively. Functional analysis revealed that the identified DEGs in the ovaries were involved in steroid hormone biosynthesis, lipid metabolism, and meiotic arrest and recovery. Besides these DEGs, the PI3K-Akt, Wnt, TGF-β, and GnRH signaling pathways have been identified as closely associated with the development and maturation of greater amberjack ovaries. These findings provide a theoretical basis for studying the molecular mechanisms underlying the oogenesis growth and maturation in greater amberjack, as well as the artificial propagation for this species.

## Figures and Tables

**Table 1:** RNA-seq data and mapping statistics of the greater amberjack ovary transcriptome.

Sample	Raw Reads	Clean Reads	Q20 (%)	Q30 (%)	GC (%)	Unique_ Mapped	Multiple_ Mapped	Total_ Mapped
FII-1	46,712,628	46,578,780	97.90	93.88	50.17	89.92%	6.12%	96.04%
FII-2	48,756,308	48,594,360	97.58	93.20	49.69	90.04%	5.53%	95.58%
FII-3	48,180,720	48,024,268	97.65	93.34	50.25	89.95%	5.95%	95.91%
FⅢ-1	48,835,906	48,675,068	97.71	93.49	49.93	90.50%	5.22%	95.72%
FⅢ-2	56,750,310	56,548,516	97.64	93.27	49.58	90.89%	5.03%	95.92%
FⅢ-3	52,184,252	52,019,122	97.66	93.37	49.68	90.54%	5.05%	95.59%
FⅣ-1	51,845,668	51,689,044	97.81	93.68	48.76	91.14%	4.98%	96.12%
FⅣ-2	38,087,518	37,954,794	97.53	93.02	49.52	90.91%	4.62%	95.53%
FⅣ-3	42,647,088	42,509,416	97.75	93.58	49.42	91.36%	4.44%	95.81%

1, 2, and 3: Three independent biological replicates. Q20: The percentage of bases with a Phred value > 20. Q30: The percentage of bases with a Phred value > 30. Unique Mapped: The number of clean reads that uniquely mapped onto the annotated genome. Multiple Mapped: The number of clean reads that mapped to multiple positions on the annotated genome. Total Mapped: The number of clean reads that mapped onto the annotated genome.

## Data Availability

The data that support the findings of this study are available upon reasonable request. The raw reads used in this article have been deposited into the Sequence Read Archive (SRA) of the NCBI database under BioProject accession number: PRJNA1138641.

## Supplementary Materials

The following supporting information can be downloaded at: https://www.mdpi.com/article/10.3390/ani15030333/s1, Figure S1. Changes in ovarian GSI in greater amberjack. FII: stage II (*n* = 3), FIII: stage III (*n* = 3), FIV: stage IV (*n* = 3); Different lowercase letters (a, b) above the error bar indicate significant differences at *p* < 0.05 in ovarian GSI at different developmental stages, as determined by one-way analysis of variance (ANOVA) and Duncan’s post-hoc test. Figure S2. Principal component analysis (PCA) showing the differences between biological groups at different stages of ovarian development. Figure S3. Validation of the expression of 8 up-regulated and 5 down-regulated DEGs using qPCR. Table S1. Sample measurement information. Table S2. Primer sequences used in this study. Table S3. Top ten up and down-regulated DEGs of ovaries in greater amberjack. Table S4. GO analysis of DEGs in the FII vs. FIII, FIII vs. FIV, and FII vs. FIV comparison groups. Table S5. DEGs of significantly enriched KEGG-Categories at different ovarian development stages. Table S6. Gene expression at different stages.
