# Supplementary material for: Transcriptomic Analysis Provides New Insights into Oocyte Growth and Maturation in Greater Amberjack (Seriola dumerili)
Source: Animals (Basel). 2025 Jan 24;15(3):333. doi: 10.3390/ani15030333 (PMC11815777; doi:10.3390/ani15030333)
Supplement: Supplementary file 1 [file animals-15-00333-s001.zip › Supplementary Figures S1-S3.pdf]

Supplementary Materials

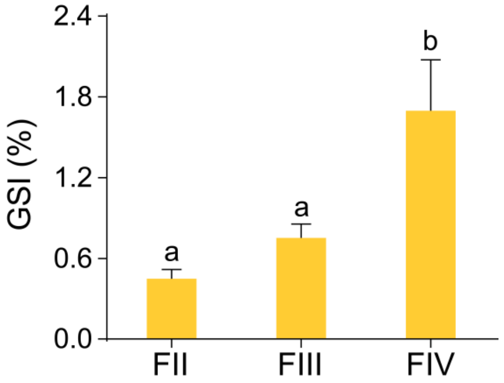

Figure S1. Changes in ovarian GSI in greater amberjack. FII: stage II (n=3), FIII: stage III (n=3), FIV: stage IV (n=3); Different lowercase letters (a, b) above the error bar indicate significant differences at $P < 0.05$ in ovarian GSI at different developmental stages, as determined by one-way analysis of variance (ANOVA) and Duncan's post-hoc test.

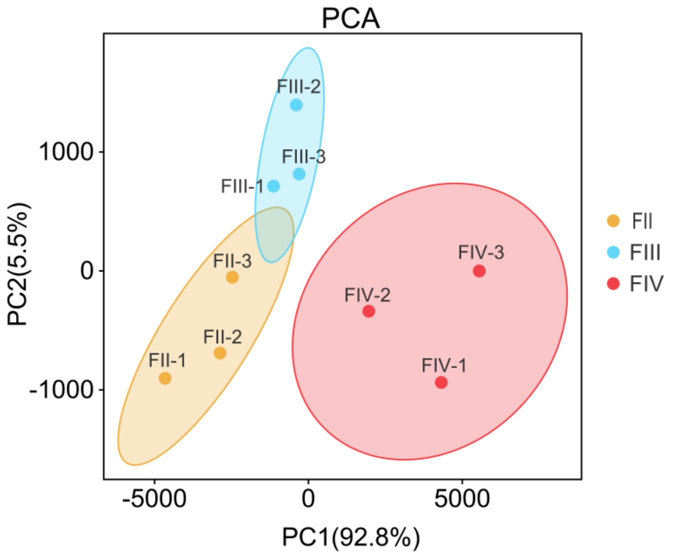

Figure S2. Principal component analysis (PCA) showing the differences between biological groups at different stages of ovarian development.

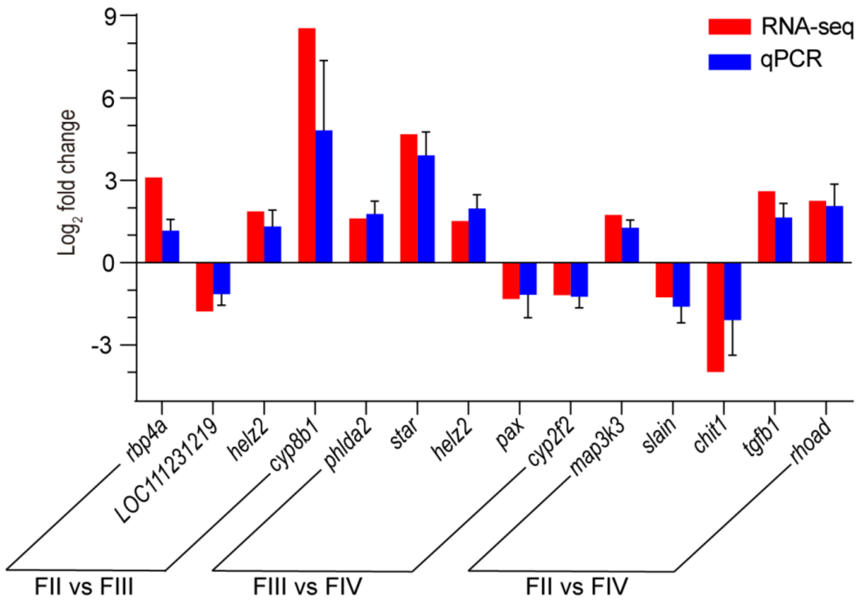

Figure S3. Validation of the expression of 8 up-regulated and 5 down-regulated DEGs using qPCR
